# Plant and Animal-Type Feedstuff Shape the Gut Microbiota and Metabolic Processes of the Chinese Mitten Crab *Eriocheir sinensis*

**DOI:** 10.3389/fvets.2021.589624

**Published:** 2021-01-26

**Authors:** Xiaowen Chen, Deyin Lu, Zhihua Li, Wucheng Yue, Jun Wang, Xiaoyan Jiang, Hua Han, Chenghui Wang

**Affiliations:** ^1^School of Medicine, Tongji University, Shanghai, China; ^2^Key Laboratory of Freshwater Aquatic Genetic Resources, Ministry of Agriculture, Shanghai Ocean University, Shanghai, China; ^3^National Demonstration Center for Experimental Fisheries Science Education, Shanghai Ocean University, Shanghai, China; ^4^Shanghai Engineering Research Center of Aquaculture, Shanghai Ocean University, Shanghai, China

**Keywords:** aquatic plants, gut microbiome communities, feed type, metabolome, *Eriocheir sinensis*

## Abstract

In animals, growth and development are strongly correlated with the gut microbiota and metabolic profiles. In this study, gut microbiome communities, metabolic profiles, and growth performance of *Eriocheir sinensis* under three dietary feed types based on waterweed plants only, freshwater snails only, and waterweed plants combined with freshwater snails were studied by using 16S rRNA gene sequencing and liquid chromatography-mass spectrometry. Results indicated that different feed types dramatically affected the growth performances of *E. sinensis* by altering the gut microbiota and metabolic profiles. Aquatic plants, such as waterweeds, played essential roles in shaping gut microbiome communities, and the optimal Bacteroides-to-Firmicutes ratio might strongly promote growth performance. Waterweed plants also helped decrease maleficent Proteobacteria caused by excess animal-type feedstuff, such as freshwater snails, and might have positive roles in antibacterial functions in gut. A diet based on waterweeds only resulted in lipid metabolism disorders, which significantly retarded the growth of *E. sinensis*. In summary, *E. sinensis* cultured with a diet of waterweeds and freshwater snails showed superior growth performance due to their healthy gut microbiota and metabolic homeostasis. Our findings unveiled the roles of aquatic plants and animal-type food such as freshwater snail in shaping the gut microbiota and metabolic processes and provided guidance for the aquaculture of *E. sinensis* in future.

## Introduction

The growth and development of animals are strongly associated with optimal and nutritious food resources. Different food resources act on animal growth and development by shaping the gut microbiome and adjusting the metabolic processes of animals (1, 2). Diverse microbiome communities in the guts of organisms provide important enzymatic resources that can be utilized for food digestion and absorption to generate the nutrients required for growth and development (3). Gut microbiomes in animals are dramatically shaped and adjusted by the changes in feed resources obtained from daily diets (1, 4). In wild captured woodrats, natural diets maintained 90% of gut microbiome communities, whereas artificial diets caused a loss of 38% of gut microbiome communities (5). In yaks, the abundance and composition of gut microbiome communities under a concentrate-feed diet with reduced crude fiber content was different from that under a common forage diet, and resulted in different growth performance (6). In pigs, fermented corn-soybean meal altered gut microbiome communities and enhanced growth performance (7). In addition to growth and development (8–10), healthy gut microbiome communities are also important for immune responses (11, 12) and the nutritional and flavor quality of specific domesticated animals (13). The metabolome refers to the complete set of small metabolites in specific organisms that reflect ongoing metabolic processes; it can be used to characterize the growth and developmental states of organisms (14). Researches have also pointed out the correlation between gut microbiome communities and metabolic profiles, which can affect the growth and development of animals (6, 15).

The Chinese mitten crab (*Eriocheir sinensis*) is an economically cultured aquatic crustacean in China (16, 17). Feed types are vital for the growth and development of *E. sinensis* during farming (18). Dietary protein and lipid sources are crucial for the growth, gonadal development, and reproduction of *E. sinensis* (19, 20). Fructooligosaccharide supplementation in the daily diet significantly affected the growth performances and antioxidant capabilities of *E. sinensis* (21). L-tryptophan supplementation in the diet improved the survival of *E. sinensis* (22). Sufficient levels of cholesterol and other related lipids benefit molting and growth (23). Many farmers have attempted to improve dietary protein/lipid contents by feeding *E. sinensis* with small animals, such as fish, shrimp, and snails, during the aquaculture process (17). Freshwater snail are the common animal-type food resources in the culture of *E. sinensis* (24). Besides, experienced fish farmers know that aquatic plants are required to culture *E. sinensis* well and healthy (17). Previous studies have indicated the numerous advantages of aquatic plants in the culture process; for example, aquatic plants adjust pH, serve as food resources, and provide shelter (25–27). Obviously, both animal-type and plant-type food resources are required for the culture of *E. sinensis* (17). However, research on the effect of aquatic plants and animal-type food such as freshwater snail on the gut microbiomes and metabolic processes of *E. sinensis* is limited.

A comprehensive analysis of the composition and dynamics of the gut microbiome will offer important insights into microbially mediated metabolic processes and help improve the efficiency and effectiveness of *E. sinensis* farming. In this study, the effects of animal-type and plant-type feedstuffs on the gut microbiomes and metabolic processes of *E. sinensis* were compared. Three diverse feed types were provided as the daily diets of *E. sinensis*: an animal-only diet that was based on freshwater snails (*Sinotaia quadrata*); a plant-only diet that was based on waterweed plants (*Elodea canadensis*); and a mixed diet of *S. quadrata* and *E. canadensis*. Then, the gut microbiomes and metabolic profiles under different feed types were obtained and compared to reveal the function of waterweed plants and freshwater snails in shaping the gut microbiomes, metabolic processes, and growth performances of *E. sinensis*.

## Materials and Methods

### Sample Collection and Ethics Statement

This study was approved by the Institutional Animal Care and Use Committee of Shanghai Ocean University (Shanghai, China). Sampling procedures complied with the guidelines of the Institutional Animal Care and Use Committee on the care and use of animals for scientific purposes. Juvenile *E. sinensis* individuals (~7.5 g) in intermolt stage were collected from the Aquatic Animal Germplasm Station of Shanghai Ocean University (Shanghai, China) and cultured in circulating aquaculture facilities for seven days. Then, all the crab individuals were divided into three groups randomly. The mixed type (Group A) was fed with waterweed plants (*E. canadensis*) and freshwater snails (*S. quadrata*). The plant-type group (Group B) was fed with waterweed plants (*E. canadensis*). The animal-type group (Group C) was fed with freshwater snails (*S. quadrata*). A total of 84 crab individuals were cultured for each group. The culture temperature was adjusted at 26 ± 2°C for all groups. The three groups were fed with sufficient feed twice a day (9:00 a.m. and 16:00 p.m.). The whole culture period lasted two molt cycles. Growth and molting status were observed every day, and body weights were recorded after the first molting, when the carapace was hardened. When crab individuals reached the premolting stage of the next molting, 800 μl of hemolymph was extracted from crab individuals in all groups with anticoagulant potassium oxalate and stored at −80°C refrigerator. Meanwhile, the gut from each crab individual in all three groups was sampled, quickly stored in liquid nitrogen, and stored at −80°C refrigerator. Six living individuals were randomly sampled from each group for gut microbiota 16S rRNA sequencing and liquid chromatography-mass spectrometry (LC–MS) metabolomics analysis.

Body weight gain (WG), shell length gain rate (SLGR), hepatosomatic index (HSI), condition factor (CF), and molting interval (MI) were calculated by using the following formulas. MI was defined as the days between two continuous molting stages during the experiment.

WG = (weight after molting − initial weight)/initial weight × 100%

SLGR = (shell length after molting − initial shell length)/initial shell length × 100%

HSI = (final hepatopancreas weight/final body weight) ×100%

CF = (final weight/shell length^3^) × 100%

### DNA Extraction and 16S rRNA Sequencing of Gut Microbiota

Gut DNA was extracted from 18 collected samples (six for each group) with FastDNA Spin Kit for Soil in accordance with the manufacturer's protocols. DNA concentration and purity were measured by utilizing NanoDrop 2000. The V3-V4 region of 16S rRNA gene was amplified by using 338F (5′-ACTCCTACGGGAGGCAGCAG-3′) and 806R (5′-GGACTACHVGGGTWTCTAAT-3′) primers. Paired-end sequencing libraries (PE300) were constructed and sequenced on an Illumina MiSeq platform (Illumina, San Diego, USA).

### Microbiome Sequence Data Processing and Analysis

After sequencing, raw sequencing reads were first quality filtered by using Trimmomatic software with the following parameters (28): Reads with an average quality score below 20 in a 50 bp sliding window were trimmed, and reads with quality below 20 at the end were also removed. Any reads with lengths of <50 bp were excluded from further analysis. After filtering, paired-end reads were merged by using FLASH with overlaps longer than 10 bp (29). Operational taxonomic units (OTUs) were clustered by applying UPARSE (7.0.1090) with ≥97% similarity, and chimeras were filtered during OTU clustering by utilizing UCHIME algorithm (30). The representative sequences of each OTU were picked to annotate taxonomic information by using the Ribosomal Database Project classifier (identity threshold of 0.7) (31). The numbers of OTUs were summarized with USEARCH 7.0 (30). The alpha diversity of Sobs and Shannon indices were calculated by applying Mothur 1.30.2 software implemented in Majorbio I-Sanger Cloud Platform (http://www.i-sanger.com) (32). Abund_jaccard distance matrixes were used to calculate beta diversity and were visualized via principal co-ordinate analysis (PCoA). Analysis of similarities (ANOSIM) was conducted to detect differences between groups by using abund_jaccard distance. PICRUSt2 was applied to predict the functions of an OTU against a database of 16S rRNA gene sequencing (33).

### LC-MS Metabolomics Processing

A total of 18 samples were subjected to LC-MS for metabolomics analysis. A total of 100 μl extracted hemolymph was mixed with 400 μl biochemical solution (acetonitrile:methanol = 1:1). Then, each sample was vortexed, ultrasonically extracted, incubated at −20°C for 30 min, and centrifuged at 13,000 rpm for 15 min at 4°C. Next, the supernatant was extracted and dried. A total of 180 μl 50% acetonitrile solution was used to redisolve dried samples for LC-MS analysis. The LC-MS experiment was conducted with an UPLC-TripleTOF platform (AB SCIEX).

### Metabolomics Data Analysis

Raw data generated through LC-MS analysis was processed with Progenesis QI (Waters Corporation, Milford, USA) for peak picking, peak alignment, peak filtering, and quantitation for each metabolite. Then, the data matrix for retention time, M/Z, and peak intensity were normalized under the following parameters: (1) only the metabolites present in > 50% of all the samples were retained; (2) missing values were replaced with 1/2 of the minimum value; and (3) peak intensities were normalized to the total spectral intensity. Then, normalized data were used to obtain accurate qualitative and quantitative results for each metabolite by matching against the HMDB (http://www.hmdb.ca/) and Metlin (https://metlin.scripps.edu) public database (34).

Positive and negative data were imported into ROPLS v1.6.2 software package (35). Orthogonal partial least squares discriminant analysis (OPLS-DA) was conducted to visualize metabolic alterations among experimental groups after mean centering and unit variance scaling. Variable importance in the projection (VIP) was used to rank the overall contribution of each variable to the OPLS-DA model, and variables with VIP > 1.0 were considered relevant for group discrimination. Metabolites with VIP > 1 and *P* < 0.05 were considered differential metabolites between groups. Differential metabolites were subjected to KEGG enrichment analysis by using software implemented in Majorbio I-Sanger Cloud Platform with corrected *P* < 0.05.

### Correlation Analysis

Pearson correlation coefficients between metabolite abundance and phenotype characteristics, between microbiome communities and phenotype characteristics, and between metabolite abundance and microbiome communities were calculated and plotted by using the corrplot package in R.

## Results

### Growth Performances Under Different Feeding Regimes

**Figure 1:**
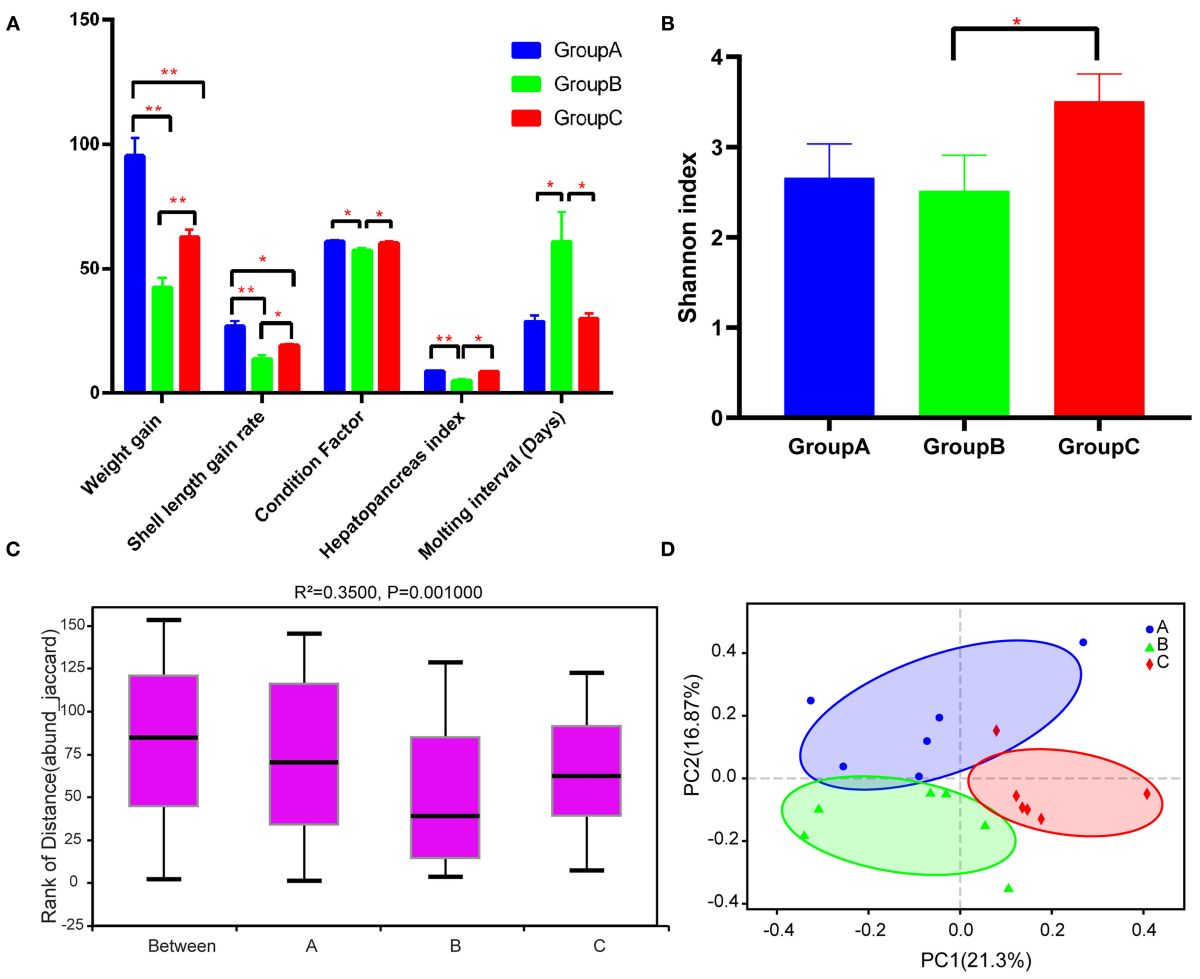
Growth performance and diversity analysis of the gut microbiota of the three groups. **(A)** Growth performance of the three groups. **(B)** Alpha diversity (Shannon index) estimate at the genus level in the three groups. **(C)** Beta diversity (ANOSIM) estimate at the OTU level in the three groups. **(D)** Beta diversity (PCA) estimates for the bacterial communities in the three groups. Note: *P < 0.05, **P < 0.01.

Group A showed significantly higher WG and SLGR than the other two groups after the experiment (*P* < 0.05) (Figure 1A). Group B had significantly lower CF and HSI than groups A and C, and groups A and C did not show significant differences in terms of these indexes (Figure 1A). Besides, group B had longer MI than groups A and C (Figure 1A).

### Sequencing, Richness, and Diversity Estimates of Microbiomes Under Different Feed Types

A total of 1,022,095 raw reads and an average of 56,783 raw reads were generated after the sequencing of each sample. A total of 1,929 OTUs were identified after filtering. The Shannon rarefaction curve between the number of reads and the Shannon index at OTU level revealed a tendency for plateau saturation for each group (Supplementary Figure 1). The alpha diversity of the sobs index indicated no significant differences among groups (*P* > 0.05), although the sobs index of group A was lower than that of groups B and C (Supplementary Figure 2). Group C had a significantly higher Shannon index than group B, indicating that gut microbial community diversity was considerably higher in group C than in group B (Figure 1B). ANOSIM analysis indicated significant differences among the three groups (*R*^2^ = 0.3500 and *P* = 0.001) (Figure 1C). Beta diversity analysis through PCoA also indicated that group C was significantly different from groups A and B (Figure 1D).

### Bacterial Community Compositions Under Different Feed Types

**Figure 2:**
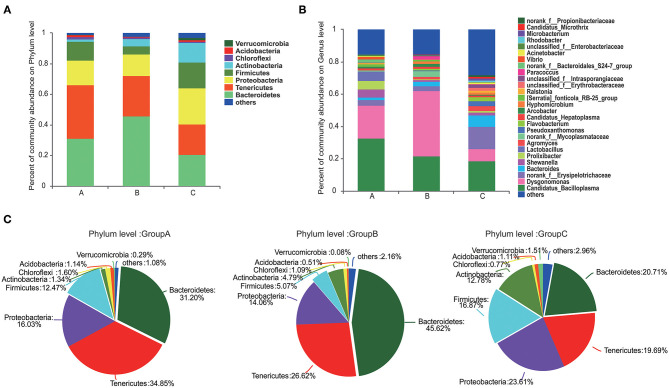
Composition of the microbiome communities of the three groups at the phylum and genus levels. **(A)** The compositions and abundances of the microbiome communities at phylum level in the three groups. **(B)** The compositions and abundances of the microbiome communities at genus levels in the three groups. **(C)** Detail information of the compositions of the microbiome communities at phylum level in the three groups.

At the phylum level, Tenericutes (34.85%) and Bacteroidetes (31.20%) were the most predominant phyla in group A. Bacteroidetes (45.62%) was the predominant phyla in group B, and Proteobacteria (23.61%) was the most predominant phyla in group C (Figure 2A). Compared with those in groups A and C, the abundance of Bacteroidetes was highest and that of Firmicutes was lowest in group B (Figures 2A,C). The abundances of Proteobacteria, Actinobacteria, and Verrucomicrobia were highest in group C.

**Figure 3:**
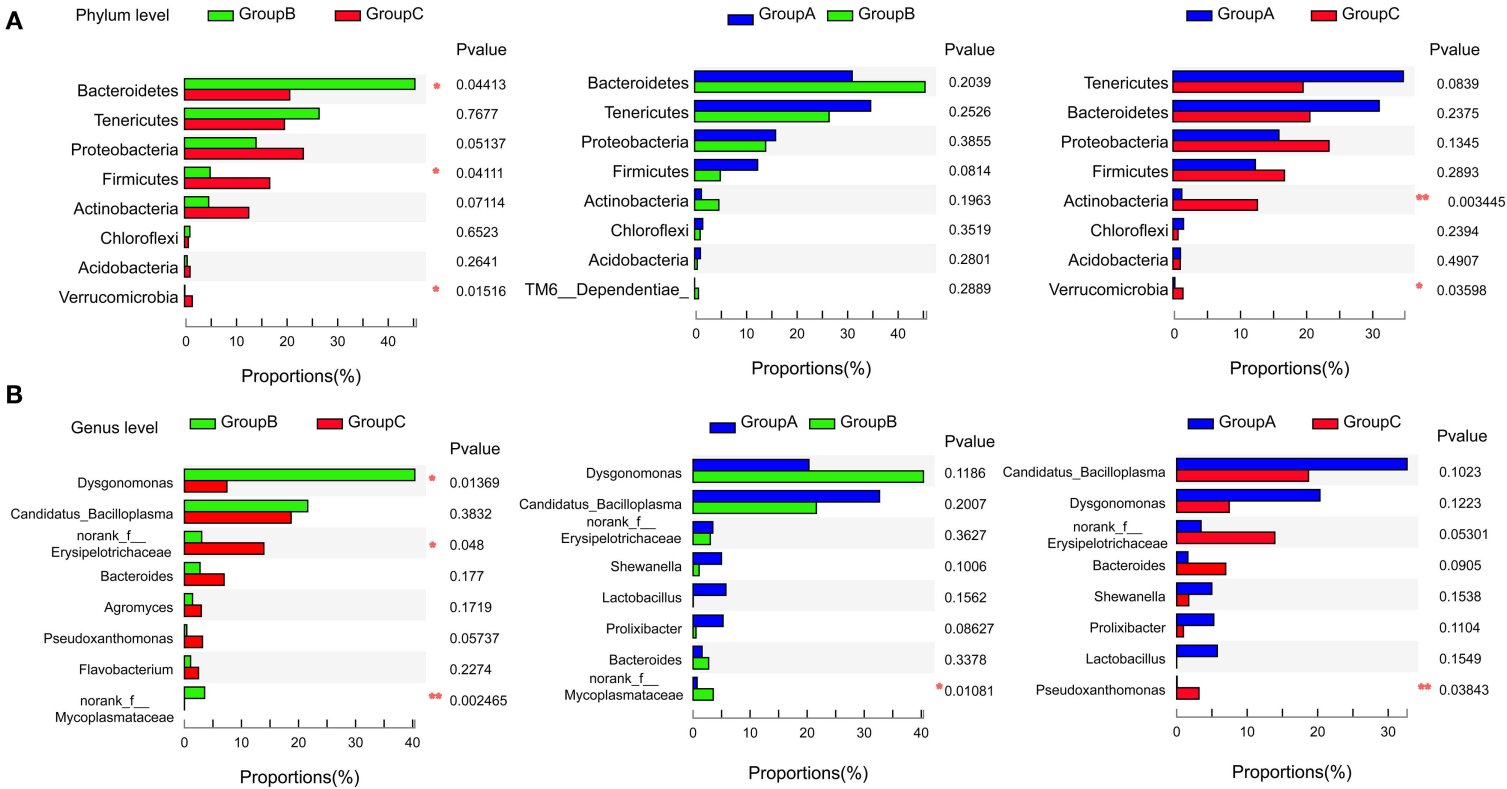
Pairwise comparison of the microbiome communities of the three groups at the phylum **(A)** and genus **(B)** levels. *P < 0.05, **P < 0.01.

Gut microbiome communities did not significantly differ between groups A and B; however, the abundance of Bacteroidetes decreased and that of Firmicutes increased in group A relative to those in group B (Figure 3A). Similarly, the abundance of Bacteroidetes was lower and that of Firmicutes was higher in group C compared with those in group B. In group B, the proportions of Bacteroidetes and Firmicutes were 45.62% and 5.07%, respectively. However, the proportion of Bacteroidetes decreased to 31.20% in group A and to 20.71% in group C, and the proportion of Firmicutes increased to 12.47% in group A and to 16.87% in group C. Bacteroidetes/Firmicutes showed a fold change from 9.00 in group B to 2.50 in group A and to 1.23 in group C (Figures 2A,C, 3A).

At the genus level, *Candidatua_Bacilloplasma* (32.68%) and *Dysgonomonas* (20.32%) were two predominant genera in group A (Figure 2B, Supplementary Figure 3). *Dysgonomonas* (40.38%) was the predominant genus in group B. *Candidatua Bacilloplasma* (18.70%) and *Erysipelotrichaceae* (13.92%) were the two predominant genera in group C (Figure 2B, Supplementary Figure 3). *Dysgonomonas* was significantly higher in group B than in group C (*P* < 0.05). The abundance of *Erysipelotrichaceae* was higher in group C than in other groups (Figure 3B).

The different functional profiles of gut microbial communities in the three groups were predicted on the basis of 16S rRNA marker gene sequences. Microbial communities in group B were predominately associated with carbohydrate metabolism and glycan biosynthesis (Supplementary Figure 4). Lipid metabolism, replication and repair, and infectious disease were the main functions of microbial communities in group C (Supplementary Figure 4).

### Comparison of Metabolomes Under Different Feed Types

**Figure 4:**
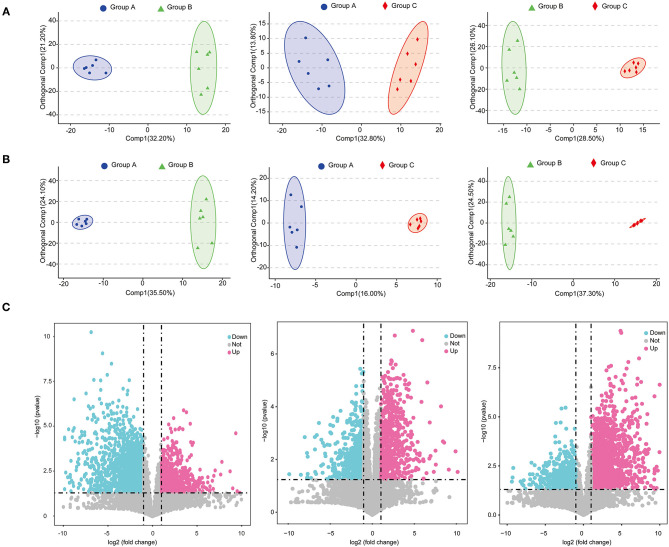
Score plots of OPLS-DA in positive mode **(A)** and negative mode **(B)**; volcano plots of metabolites identified between groups with fold change and *P*-value presented **(C)**.

A total of 6,146 metabolites (positive-ionized mode) and 4,055 metabolites (negative-ionized mode) were identified in this study. After annotation, we obtained 411 known metabolites. The score plots of the OPLS-DA were generated to present a global overview of the differences in metabolites among the three feed groups. Positive and negative data revealed clear separation and discrimination among the three groups (Figures 4A,B).

A total of 621 differential metabolites were identified, among which 240 were up-regulated and 381 were down-regulated in group B (positively ionized and negatively ionized mode) relative to in group A (Figure 4C). In groups A and C, 514 differential metabolites were identified, among which 262 were up-regulated and 252 were down-regulated in group C (positively ionized and negatively ionized mode) (Figure 4C). In groups B and C, 685 differential metabolites were identified, among which 375 were up-regulated and 310 were down-regulated in group C relative to in group B (positively ionized and negatively ionized mode) (Figure 4C).

**Figure 5:**
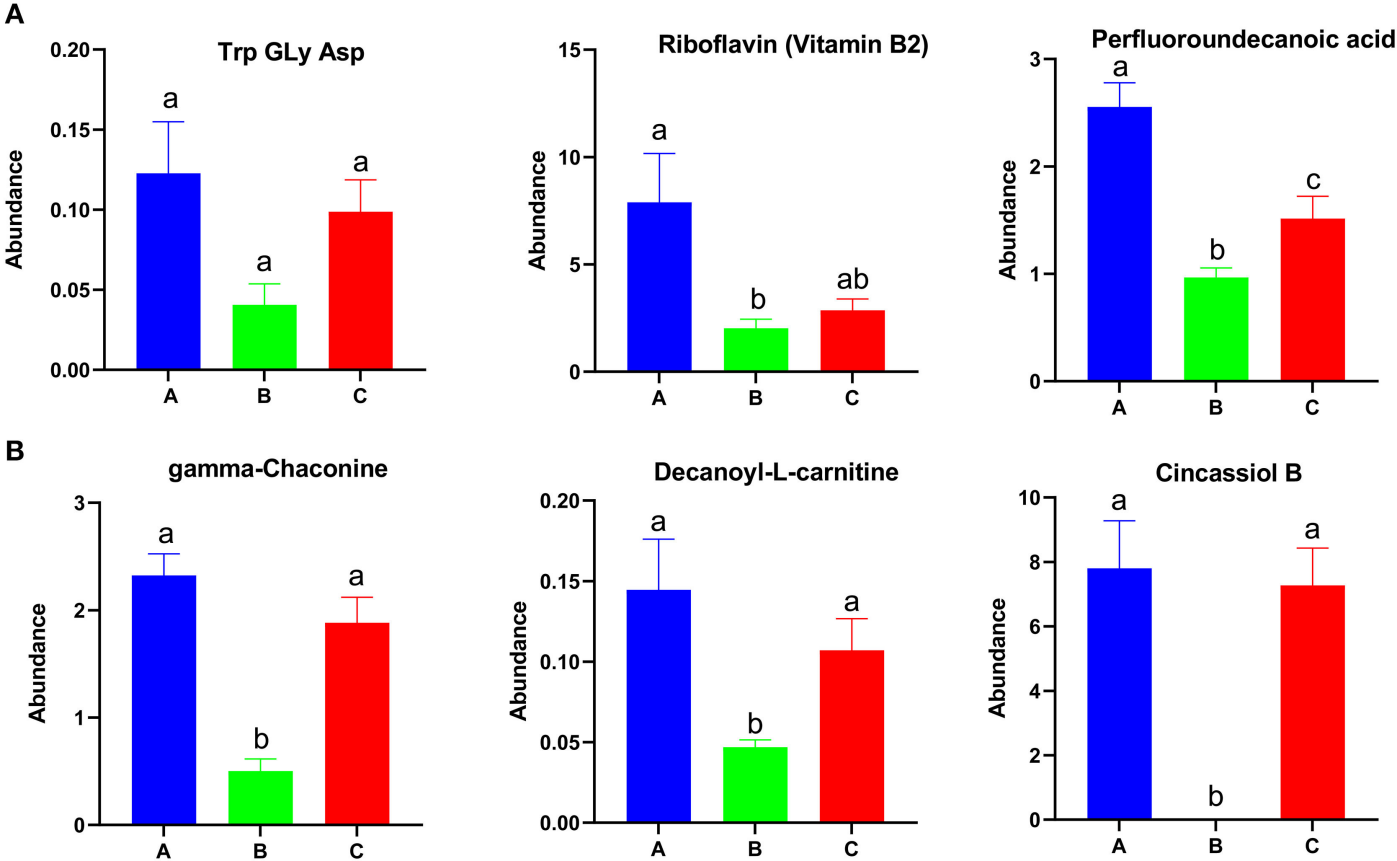
Identified differential metabolites among the three feed groups with *P* < 0.05. Different letters represent significant differences, and same letters represent no significant differences.

Among the differential metabolites, 53 were up-regulated and 25 were down-regulated in group A relative to in groups B and C (Supplementary Figure 5A). A total of 166 metabolites were up-regulated and 239 metabolites were down-regulated in group B relative to in groups A and C (Supplementary Figure 5B). A total of 94 metabolites were up-regulated and 89 metabolites were down-regulated in group C relative to in groups A and B (Supplementary Figure 5C). The abundances of Try, Gly, Asp, riboflavin (vitamin B2), and perfluoroundecanoic acid were higher in group A than in other groups (Figure 5A). The abundance of gamma-chaconine, decanoyl-L-carnitine, and cincassiol B were lowest in group B (Figure 5B). MG, norvitamin D3, and L-a-Lysophosphatidylserine were highest in group B and were enriched in autophagy and the glycosylphosphatidylinositol-anchor biosynthesis pathway (Supplementary Figure 6). Different types of glyceryl phosphatide metabolites, such as PS, PE, LysoPC, and LysoPE, were up-regulated in group C and were enriched in glycerophospholipid metabolism, choline metabolism in cancer, and the retrograde endocannabinoid signaling pathway (Supplementary Figure 7).

### Correlation Analysis Between Growth Performance Indexes, Microbiome Profiles, and Metabolites

**Figure 6:**
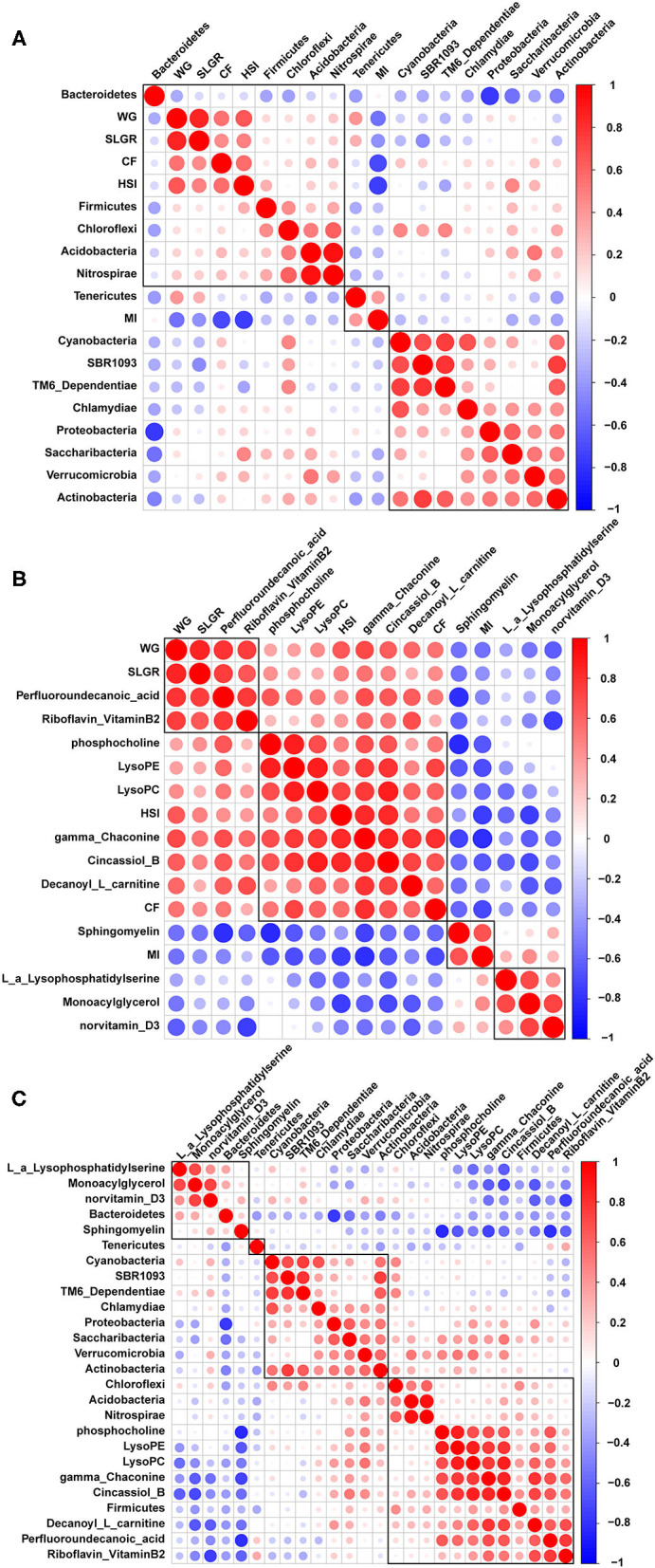
Pearson correlation analysis between growth performance indexes and gut microbiota **(A)**, between growth performance indexes and related differential metabolites **(B)** and between gut microbiota and related differential metabolites **(C)**.

Correlation analysis between growth performance indexes and the top 14 microbiome communities at the phylum level indicated that WG, SLGR, CF, and HSI were positively correlated with Firmicutes, Acidobacteria, and Nitrospirae. MI was positively correlated with Tenericutes (Figure 6A). Correlation analysis between growth performance indexes and differential metabolites were also conducted and indicated that WG and SLGR were positively correlated with the abundance of riboflavin (vitamin B2). CF and HSI were positively correlated with the abundance of gamma-Chaconine, cincassiol B, and decanoyl-L-carnitine (Figure 6B). MI was positively correlated with Sphingomyelin. Meanwhile, correlation analysis between microbiome communities and differential metabolites indicated that riboflavin (Vitamin B2), gamma-Chaconine, cincassiol B, and decanoyl-L-carnitine were positively correlated with Firmicutes, Acidobacteria, and Nitrospirae (Figure 6C).

## Discussion

In this study, the gut microbiome communities and metabolic profiles under three feed types were characterized. Different growth performances were presented accompanied with diverse gut microbiome compositions and metabolic profiles, indicating that feed types dramatically affected the growth and development of *E. sinensis*. An optimal daily diet can shape a healthy gut microbiome and benefit the metabolic processes of *E. sinensis*, leading to superior growth performance during aquaculture.

Previous studies indicated that Tenericutes, Proteobacteria, Bacteroidetes, and Firmicutes were the predominant microbiome communities in the guts of juvenile *E. sinensis* (18). This observation was consistent with the microbiome community composition of group A, which received a diet of freshwater snails and waterweed plants (Figure 2). This result indicated that a daily diet similar to the diet of group A might be the best fit for *E. sinensis*. The growth performance of *E. sinensis* in group B was significantly inferior to that of groups A and C (Figure 1A). The abundances of Bacteroidetes and Firmicutes in the three groups were significantly different (Figure 2). Previous studies indicated that Bacteroidetes can produce carbohydrate metabolism-related enzymes to promote food digestion and provide nutrients to the host (36, 37). Firmicutes promotes energy acquisition by improving lipid metabolism (38, 39). Moreover, the proportion of Bacteroidetes and Firmicutes can affect the ability of intestinal bacteria to metabolize nutrients, thus affect growth and WG (40, 41). It is reported that a low proportion of Bacteroidetes and a high proportion of Firmicutes was existed in the gut microbiomes of obese animals (42–44). In the present study, we identified that the ratio of Bacteroides-to-Firmicutes increased in group B than in groups A and C, which may affect nutrients metabolism and restrain the growth of *E. sinensis*. However, a low Bacteroides-to-Firmicutes ratio was identified in group A, which may explain the superior growth in group A than group B. Although studies have indicated that a low Bacteroides-to-Firmicutes ratio will benefit growth, whether a lower Bacteroides-to-Firmicutes ratio provides more benefits than a higher ratio and whether an optimal Bacteroides-to-Firmicutes ratio exists are unknown. In this study, the Bacteroides-to-Firmicutes ratio of groups A and C was both lower than that of group B. The ratio of group C was lower than that of group A, and the growth performance of group C was inferior to that of group A, indicating the existence of an optimal Bacteroides-to-Firmicutes ratio of the gut microbiome. Under a mixed diet of waterweed plants and freshwater snails that was provided to group A, the Bacteroides-to-Firmicutes ratio changed to 2.5. This effect might shape the gut microbiome into its best condition and strongly promote the growth performance of *E. sinensis*. However, additional detailed research needs to be conducted in the future to confirm this hypothesis.

Dietary lipids are required for the growth and development of *E. sinensis*, cholesterol and other lipids need to be obtained from the daily diet (45). Crabs in group B received nearly no lipids from their diet, and their growth was severely retarded (Figure 1A). Crabs in group C received abundant lipids and exhibited better growth performance than crabs in group B. However, the growth performance of group C was inferior to that of group A. Excessive animal-type feedstuff will result in intensive microorganism breeding and affect water quality, which will negatively affect the growth of *E. sinensis* (17). Many studies have highlighted the correlation between the gut microbiota and immunity in animals (7, 15, 46). In this study, the abundance of Proteobacteria was highest in group C (Figure 2). Members of Proteobacteria are believed to be maleficent bacteria that cause the infection of organisms, and a high level of Proteobacteria is a potential diagnostic criterion for dysbiosis and disease (46, 47). The high proportion of Proteobacteria in group C may damage the ecosystem balance of the gut. The functional prediction of the gut microbiome in group C by PICRUST2 software also indicated the presence of microbiome communities associated with immune diseases, indicating that excessive animal-type feedstuff, such as freshwater snails, might severely harm the gut of *E. sinensis* (Supplementary Figure 4) and might account for the inferior growth performance of group C relative to that of group A. Interestingly, with the provision of waterweed plants to the freshwater snail diet (group A), the abundance of Proteobacteria decreased, and a healthy gut microbiome was shaped, thus accounting for the best growth performance shown by group A. Waterweed plants might have positive roles in antibacterial function by altering gut microbiota.

Moreover, the content of Bacteroidetes (45.62%) was the highest in group B. Bacteroidetes plays roles in lignocellulose degradation (48). The gut microbiome was shaped into an efficient microbiome community environment for plant digestion when only waterweeds plants were provided to group B. *Dysgonomonas* was the predominant genus in group B. It plays an important role in plant cell wall degradation and can digest lignocellulose (18, 49). The functional profiling of microbiome communities also indicated that the microbiome of group B was associated with glycan biosynthesis and metabolism, digestive system, and energy metabolism (Supplementary Figure 4). These results indicated that *E. sinensis* could digest and take full advantage of waterweeds plants. However, the growth performance of group B was the worst because the daily diet of this group failed to provide abundant lipids.

Our metabolome data revealed that the metabolic profiles of *E. sinensis* were also altered by feed types and might be linked with gut microbiota activities. Lipids are essential for the growth of crustaceans (45). In this study, freshwater snails were provided to groups A and C to guarantee that dietary lipid requirements were met. However, almost no extra lipids were provided to group B. Although waterweed plants have essential roles in regulating gut microbiome health, a diet of only waterweed plants was unsuitable for the growth of *E. sinensis* (Figure 1A). Differential metabolites were enriched in the autophagy pathway in group B, indicating that *E. sinensis* was severely damaged by lipid shortage and metabolic disorders (Supplementary Figures 6, 7). Correlation analysis revealed that WG, SLGR, CF, and HSI were positively correlated with cincassiol B and decanoyl-L-carnitine. Cincassiol B belongs to a class of organic compounds known as diterpenoids and plays essential roles in lipid transport and metabolism (50). L-carnitine is an necessary nutrient that plays a crucial role in the production of energy metabolism by transporting fatty acids into mitochondria (51). The amounts of these two metabolites were low, indicating that energy supplementation was insufficient in group B. Meanwhile, we also found that metabolites, such as Trp, Gly, Asp, riboflavin, and gamma-chaconine, were up-regulated in group A. These metabolites are associated with energy metabolism and immune response, indicating that the diet with waterweeds plants and freshwater snails may help with metabolic homeostasis and promote the growth of *E. sinensis* in group A in this study (52, 53).

In summary, neither waterweed plants nor freshwater snails were the optimal feed type for the growth and development of *E. sinensis*. A mixed diet containing waterweed plants and freshwater snails was the best choice. This type of diet will maintain healthy gut microbiome communities and provide sufficient nutrients, such as lipids and amino acids, to sustain metabolic hemostasis in *E. sinensis*. Our study preliminarily unveiled the function of aquatic plants in shaping gut microbiome communities and adjusting metabolic processes. However, detailed functional studies need to be conducted to further unveil the regulation and interaction of gut microbial and metabolism of *E. sinensis* in future. Our results provide guidance for the culture and feed formula design of *E. sinensis* and other crustaceans.

## Data Availability Statement

All raw sequencing sequences have been submitted to the NCBI sequences read archive database with BioProject accession number PRJNA655983.

## Ethics Statement

The animal study was reviewed and approved by Institutional Animal Care and Use Committee of Shanghai Ocean University.

## Author Contributions

CW, HH, and XC designed the study and wrote the manuscript. DL, ZL, and WY collected samples and executed experimental work. JW and XJ processed data. All authors contributed to the article and approved the submitted version.

## Conflict of Interest

The authors declare that the research was conducted in the absence of any commercial or financial relationships that could be construed as a potential conflict of interest.

## Supplementary Material

Supplementary Figure 1 Shannon rarefaction curve between read numbers and Shannon indexes at the OTU level.

Supplementary Figure 2 Alpha diversity (Sobs indexes) estimates in the three groups.

Supplementary Figure 3 The compositions and abundances of the microbiome communities of the three groups at the genus level.

Supplementary Figure 4 Functional prediction of gut microbiome communities of the three groups.

Supplementary Figure 5 Up-regulated/down-regulated metabolites identified in group A **(A)**, group B **(B)**, and group C **(C)**.

Supplementary Figure 6 Information of up-regulated metabolites identified in group B **(A)** and KEGG enrichment analysis results on the differential metabolites **(B)**.

Supplementary Figure 7 Information of up-regulated metabolites identified in group C **(A)** and KEGG enrichment analysis results on the differential metabolites **(B)**.
