# Supplementary figures and images for: Plant and Animal-Type Feedstuff Shape the Gut Microbiota and Metabolic Processes of the Chinese Mitten Crab Eriocheir sinensis
Source: Front Vet Sci. 2021 Jan 26;8:589624. doi: 10.3389/fvets.2021.589624 (PMC7870710; doi:10.3389/fvets.2021.589624)

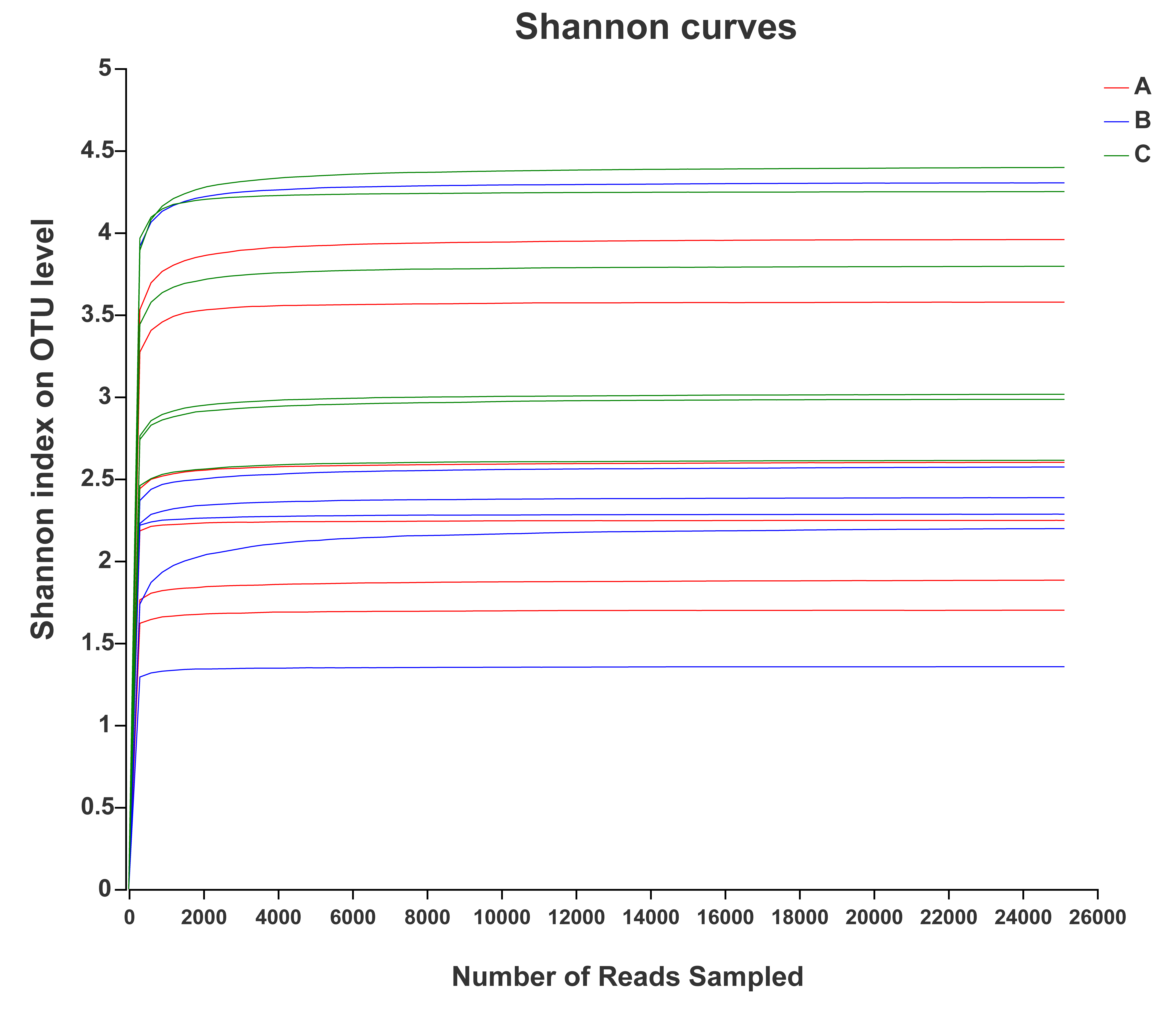

Supplement: Supplementary Figure 1 — Shannon rarefaction curve between read numbers and Shannon indexes at the OTU level. [file Image_1.TIF]

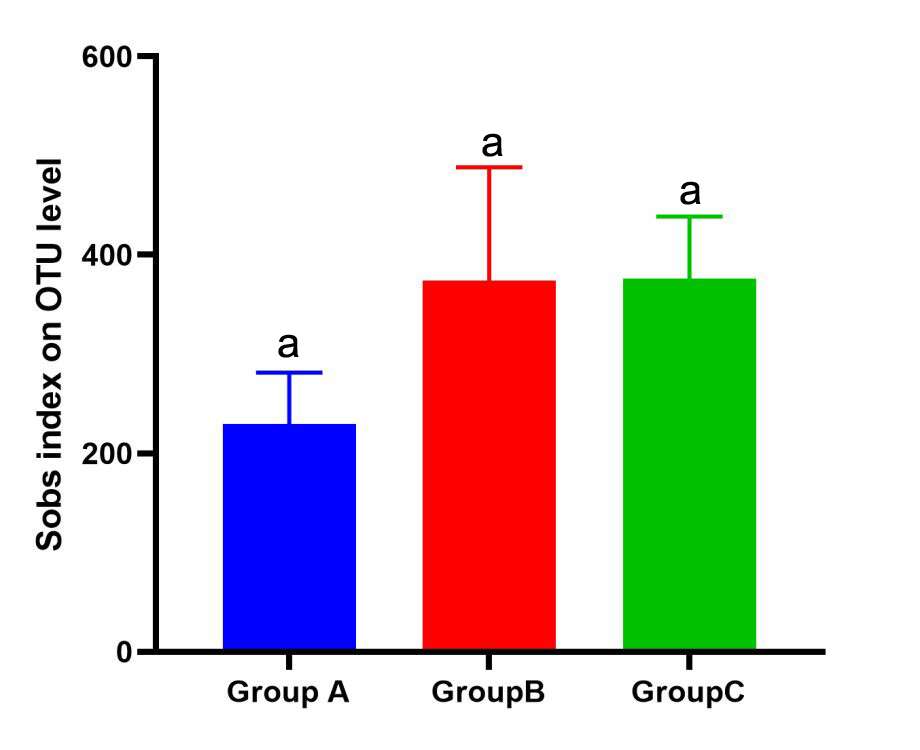

Supplement: Supplementary Figure 2 — Alpha diversity (Sobs indexes) estimates in the three groups. [file Image_2.TIF]

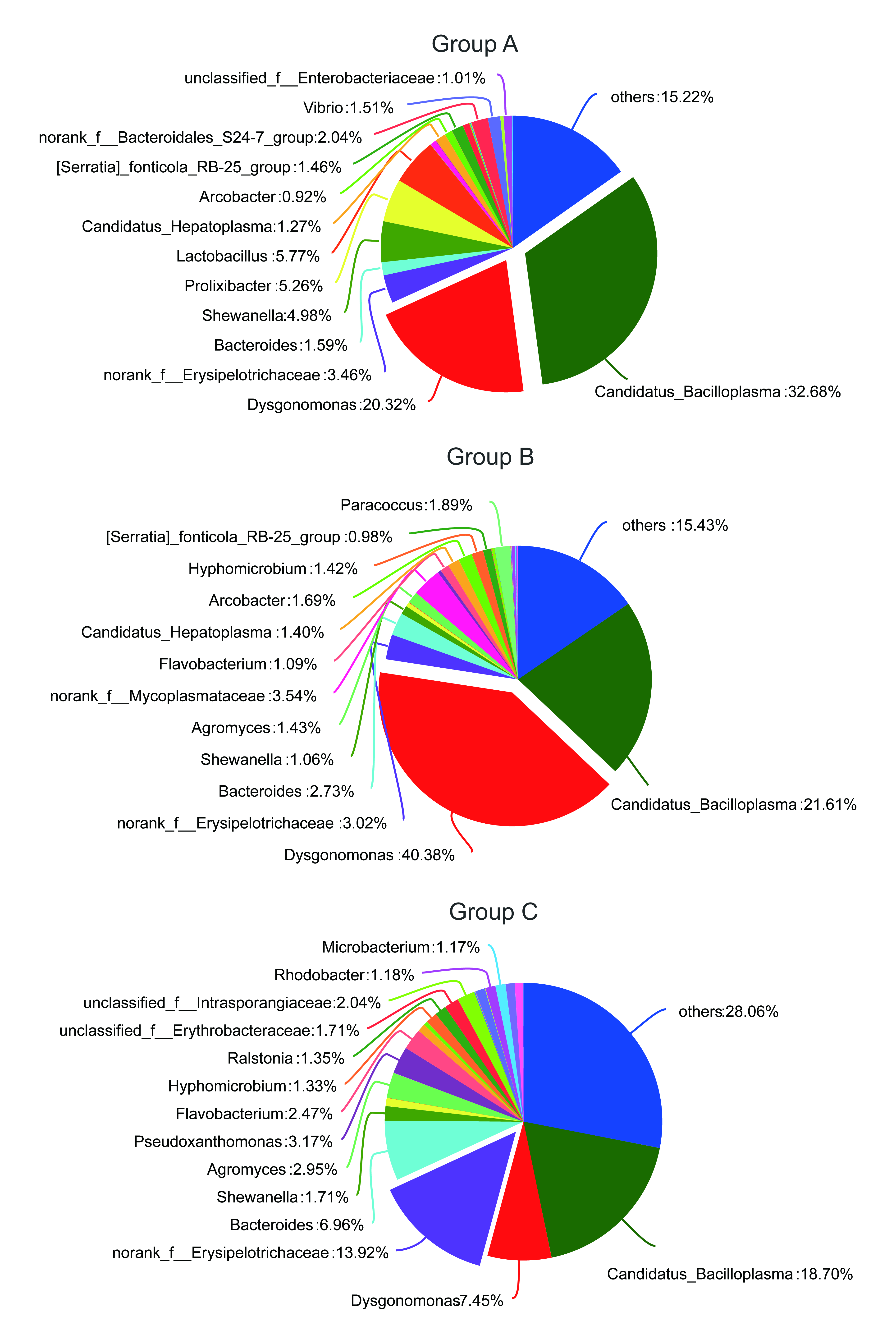

Supplement: Supplementary Figure 3 — The compositions and abundances of the microbiome communities of the three groups at the genus level. [file Image_3.TIF]

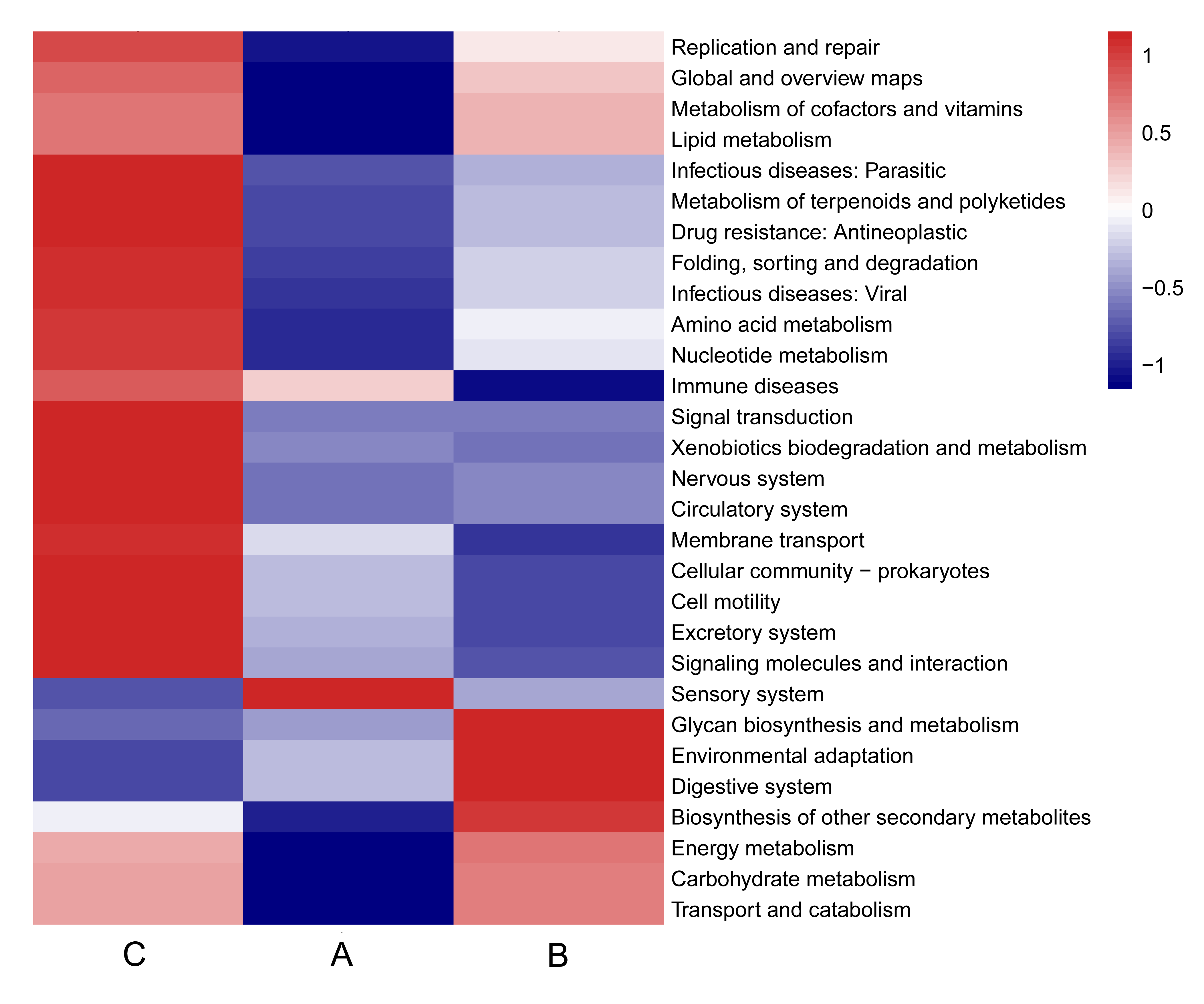

Supplement: Supplementary Figure 4 — Functional prediction of gut microbiome communities of the three groups. [file Image_4.TIF]

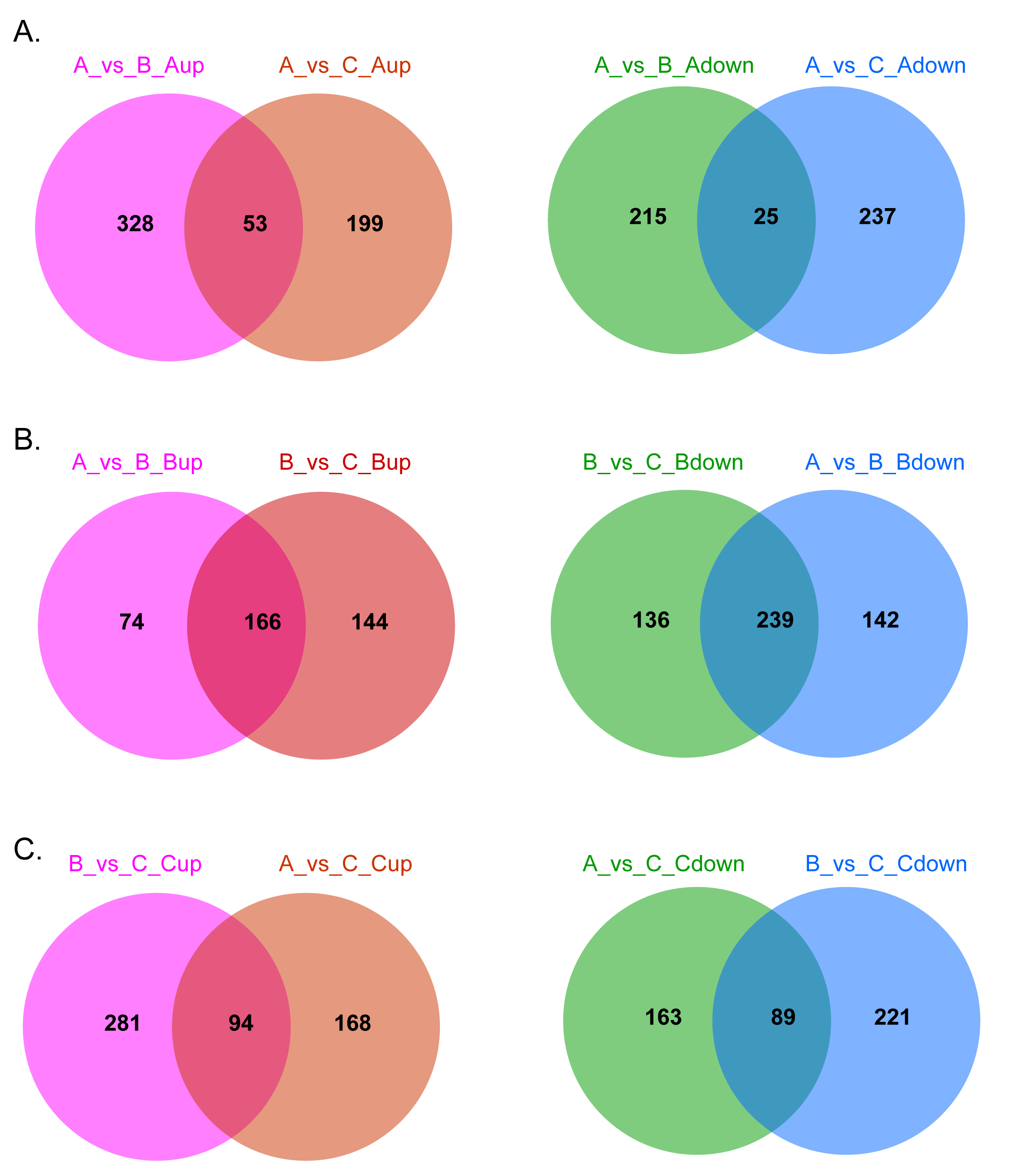

Supplement: Supplementary Figure 5 — Up-regulated/down-regulated metabolites identified in group A (A), group B (B), and group C (C). [file Image_5.TIF]

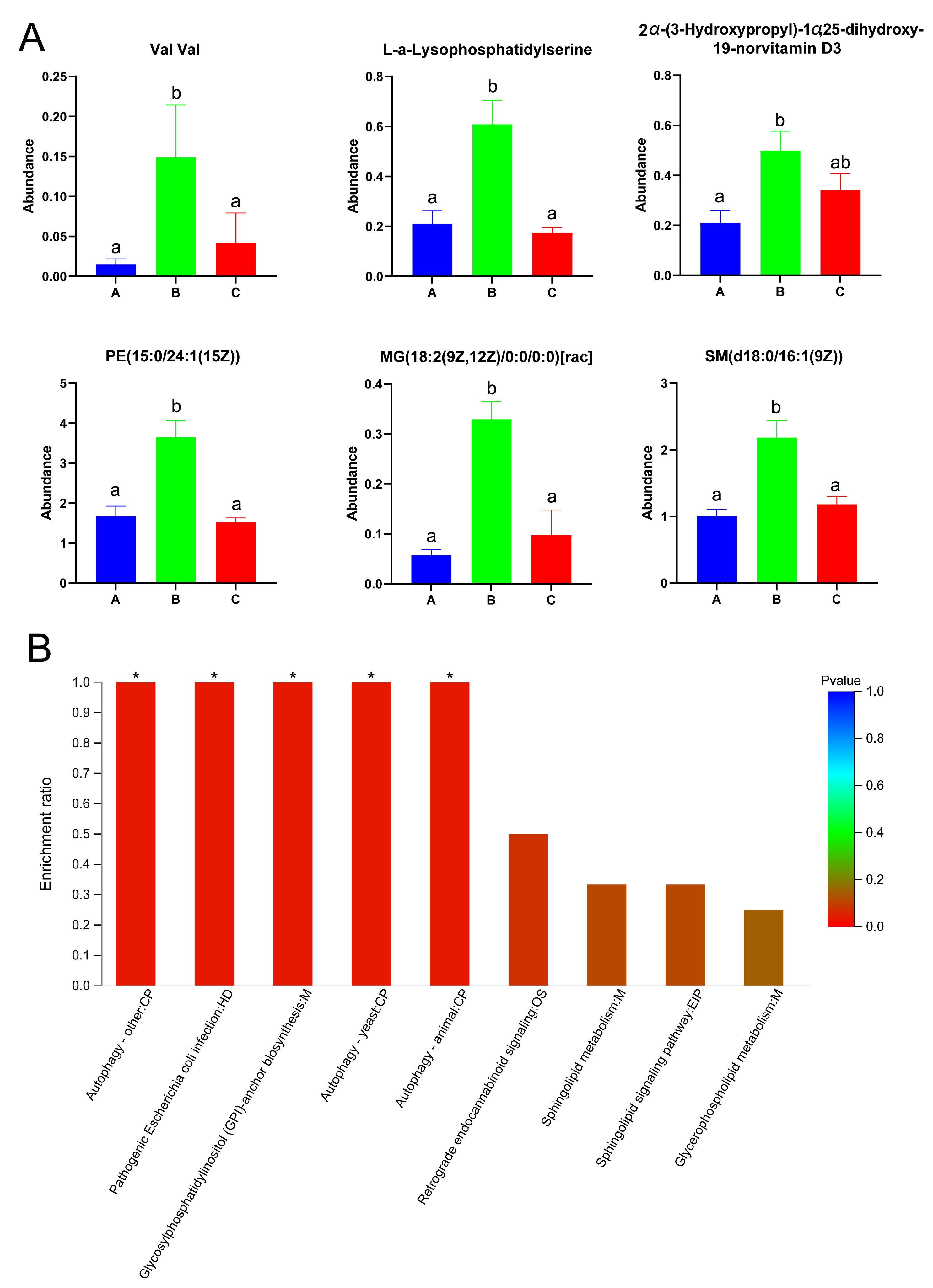

Supplement: Supplementary Figure 6 — Information of up-regulated metabolites identified in group B (A) and KEGG enrichment analysis results on the differential metabolites (B). [file Image_6.TIF]

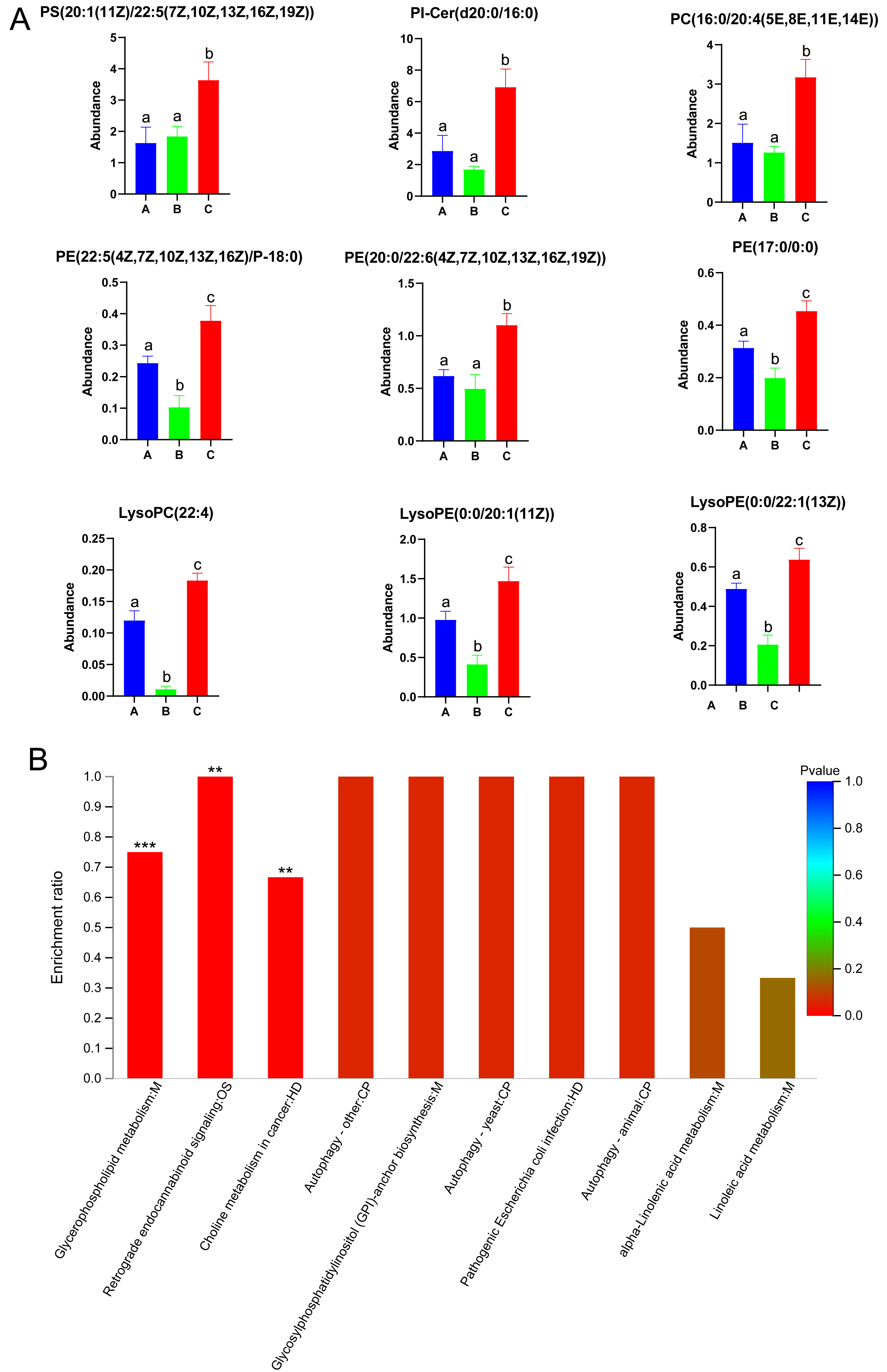

Supplement: Supplementary Figure 7 — Information of up-regulated metabolites identified in group C (A) and KEGG enrichment analysis results on the differential metabolites (B). [file Image_7.TIF]
